# Cytotoxicity, Antiproliferative Effects, and Apoptosis Induction of Methanolic Extract of *Cynometra cauliflora* Linn. Whole Fruit on Human Promyelocytic Leukemia HL-60 Cells

**DOI:** 10.1155/2012/127373

**Published:** 2012-11-04

**Authors:** T-Johari S. A. Tajudin, Nashriyah Mat, Abu Bakar Siti-Aishah, A. Aziz M. Yusran, Afnani Alwi, Abdul Manaf Ali

**Affiliations:** ^1^Department of Biotechnology, Faculty of Agriculture and Biotechnology, Universiti Sultan Zainal Abidin, Gang Badak Campus, 21300 Kuala Terengganu, Terengganu, Malaysia; ^2^Department of Agricultural Sciences, Faculty of Agriculture and Biotechnology, Universiti Sultan Zainal Abidin, Gang Badak Campus, 21300 Kuala Terengganu, Terengganu, Malaysia; ^3^Department of Animal Science, Faculty of Agriculture and Biotechnology, Universiti Sultan Zainal Abidin, Gang Badak Campus, 21300 Kuala Terengganu, Terengganu, Malaysia

## Abstract

Methanolic extract of *Cynometra cauliflora* whole fruit was assayed for cytotoxicity against the human promyelocytic leukemia HL-60 and the normal mouse fibroblast NIH/3T3 cell lines by using the MTT assay. The CD_50_ of the extract for 72 hours was 0.9 **μ**g/mL whereas the value for the cytotoxic drug vincristine was 0.2 **μ**g/mL. The viability of the NIH/3T3 cells was at 80.0% when treated at 15.0 **μ**g/mL. The extract inhibited HL-60 cell proliferation with dose dependence. AO/PI staining of HL-60 cells treated with the extract revealed that majority of cells were in the apoptotic cell death mode. Flow cytometry analysis of HL-60 cells treated at CD_50_ of the extract showed that the early apoptotic cells were 31.0, 26.3 and 19.9% at 24, 48, and 72 hours treatment, respectively. The percentage of late apoptotic cells was increased from 62.0 at 24 hours to 64.1 and 70.2 at 48 and 72 hours, respectively. Meanwhile, percent of necrotic cells were 4.9, 6.6, and 8.5 at 24, 48, and 72 hours, respectively. This study has shown that the methanolic extract of *C. cauliflora* whole fruit was cytotoxic towards HL-60 cells and induced the cells into apoptotic cell death mode, but less cytotoxic towards NIH/3T3 cells.

## 1. Introduction

*Cynometra cauliflora* (Leguminosae), a member of the bean family Fabaceae with vernacular name *nam nam*, is an indigenous plant of Malaysia that is widely distributed in South East Asia, Ceylon, and western and southern Peninsular of India [1]. This plant is a typical underutilized fruit tree that has the medicinal values in folk traditional medicine and cultivated as an ornamental plant in the village [2, 3]. This evergreen and much-branched small tree or shrub can grow up to 15 m tall with flowers and fruits on its trunk. The fruits are kidney-shaped pod, greenish-yellow to brown in colour, with a rough and wrinkled surface. Unripe fruit tastes sour and the mature fruit is also cooked with sugar to make *compote*. It can also be consumed as fruit salad (*ulam*), pickled, or be used as a condiment based on pounded chili [1]. The fruit has a large seed that was reported to have low antioxidant capacity and moderately high in total phenolic content [3]. 

*Ulam* or traditional vegetables and medicinal plants of the Malaysian forest were reportedly rich in biological activities such as antimicrobial, cytotoxic, antiviral, anti-inflammation, antioxidant, antitumor promoting, and antidiabetic activities [4–11]. Several interesting natural product compounds were isolated from local medicinal plants such as damnacanthal from *Morinda elliptica* that was reported to have anti-HIV and immunomodulating activities [12–15], goniothalamin from *Goniothalamus* spp. that was cytotoxic towards leukemic cells and also induced apoptotic cell death [16, 17], as well as phytosterols from *Coleus tuberosus*, garcinia acid esters from *Garcinia atroviridis,* and girinimbine from *Murraya koenigii* that were reported to have antitumour promoting activities [18–20]. In this paper, we reported that the crude methanolic extract of *C. cauliflora* whole fruit has a very significant cytotoxicity towards promyelocytic leukemia HL-60 cells. However, the extract was less cytotoxic towards normal mouse fibroblast NIH/3T3 cells. The extract was found to inhibit the HL-60 cells proliferation and also induced apoptotic cell death mode.

## 2. Materials and Methods

### 2.1. Plant Identification and Documentation

The fruit of *Cynometra cauliflora* was collected from the district of Kuala Terengganu, Terengganu, Malaysia, and was identified by a botanist, Dr Nashriyah Mat, Department of Agricultural Science, Universiti Sultan Zainal Abidin, Malaysia. A voucher specimen was deposited at the herbarium of the Faculty of Agriculture and Biotechnology (HUDM 354114).

### 2.2. Sample Preparation

The whole fruits of *C. cauliflora* were dried in an oven at 40°C for 5 days and then soaked in methanol for another 3 days in the dark. The solvent was removed by using a rotary evaporator under reduced pressure at a temperature of 40°C and the residue was collected. A stock solution of the extract residue with concentration of 15.0 mg/mL (w/v) was prepared in 100% dimethyl sulphoxide (DMSO) and then further diluted in RPMI-1640 free serum media to give a working stock concentration of 60.0 *μ*g/mL.

### 2.3. Cell Line

Human promyelocytic leukemia HL-60 and normal mouse fibroblast NIH/3T3 cell lines were purchased from ATCC (Manassas, VA, USA). Cells were grown in RPMI-1640 media (Sigma, St. Louis, USA), supplemented with 10% foetal bovine serum (HyClone, USA) and antibiotics (100.0 units/mL penicillin and 100.0 *μ*g/mL streptomycin) (PAA, Austria), and maintained in an incubator at 37°C with 5% CO_2_ in a humidified atmosphere. HL-60 cells were sub-cultured every 2 or 3 days by splitting the culture 1 : 1 or 1 : 2 in the new flask with fresh growth medium. For adherent cells of NIH/3T3, semiconfluent cells were treated with trypsin-like enzyme with phenol red (Gibco, USA) for 5 minutes then the cells were resuspended in the medium with serum and transferred into 2 or 3 new flasks. Cell viability of above 95% was used throughout this study.

### 2.4. MTT Cytotoxicity Assay

The MTT assay was carried out in the 96-wells plate as described by Ali et al. [6]. Briefly, a volume of 50.0 *μ*L of complete growth medium was added into each well of 96-wells flat bottom microtiter plate (Nunclon, USA). The extract or vincristine sulphate solution (95.0–105.0% purity by HPLC, Sigma, USA) at 60.0 *μ*g/mL was aliquoted into wells in triplicate and serially diluted. A volume of 50.0 *μ*L of 1-2 × 10^5^ cells/mL HL-60 or NIH/3T3 cells were seeded into 96-wells flat microtiter plates and incubated for 72 h in CO_2_ incubator. After 72 hours incubation, a volume of 20.0 *μ*L of MTT solution (5.0 mg/mL) was added into each well and incubated for 4 hours. The culture medium was removed and 100.0 *μ*L of 100% DMSO solution were added to each well to solubilise the formazan formed [21]. The plates were read using the plate reader at 570 nm with reference at 630 nm wavelength (Infinite M200, Tecan, Switzerland). A dose response curve of the percentage of cell viable versus extract concentration was plotted.

### 2.5. Cell Proliferation Assay

Briefly in this assay, HL-60 cells at the concentration of 1 × 10^5^ cells/mL were incubated with extract at CD_25_, CD_50_, and CD_75_ values for 24, 48, and 72 h in 96-wells plates. The MTT assay was performed and the absorbance of each well was read at 570 nm with reference at 630 nm wavelength as mentioned above. The proliferation graph was established by plotting the optical density (OD) values versus time. 

### 2.6. Acridine Orange and Propidium Iodide Staining (AO/PI)

HL-60 cells were treated with the extract for 24 hours at CD_50_ concentration and H_2_O_2_ was used as a positive control. Cells without treatment were used as a negative control. Each of the treatment was done in triplicate. After 24 hours incubation, the cells were harvested into centrifuge tubes and pelleted down at 100 g for 10 min. The cell pellets were washed with PBS by centrifuging the cells as mentioned above. Then the pellets were suspended in 50.0 *μ*L of acridine orange (10.0 *μ*g/mL) and 50.0 *μ*L of propidium iodide (10.0 *μ*g/mL) for 5 min. A volume of 10.0 *μ*L of stained cells was pipetted onto glass slide and covered with a cover slip. The viable, apoptotic and necrotic cells were scored in a population of 100 cells as described by Ali et al. [22] by using the fluorescent microscope (Nikon TE2000-U, Nikon, Japan).

### 2.7. Flow Cytometry Analysis-Annexin V-FITC/PI

The HL-60 cells were treated with extract at CD_50_ for 6, 24, 48, and 72 hours. After incubation, cells were harvested into 5 mL centrifuge tubes and spun down at 300 g for 10 min. Using cold PBS, the cells were washed trice and a volume of 100 *μ*L binding buffer (annexin V-FITC Apoptosis Detection Kit I, (Becton Dickenson)) was added into the tube. A volume of 1.25 *μ*L of Annexin V-FITC and 1.25 *μ*L PI solutions were added into the tube and incubated in the dark for 15 min. Then a volume of 400 *μ*L 1X binding buffer was added to each tube and gently vortexed before analyzed by using a flow cytometer (FACSCalibur, (Becton Dickenson, USA)). About 10 000 events were sorted accordingly into viable, early apoptotic, late apoptotic, and necrotic cells [23].

### 2.8. Statistical Analysis

Results were expressed as mean values with ±standard deviation of the mean. All data were performed in triplicates and analyzed using the student's *t*-test or one-way ANOVA, where differences were considered significant at *P* ≤ 0.05.

## 3. Results and Discussion

**Figure 1:**
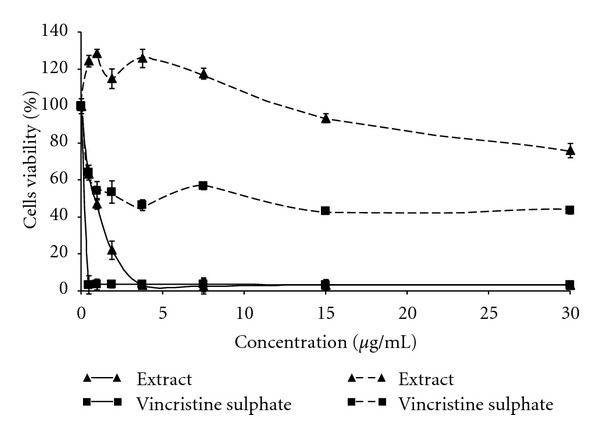
Effects of crude extract and vincristine sulphate on the viability of HL-60 cells (▲**—**▲ extract; ■**—**■ vincristine sulphate) and 3T3/NIH cells (▲- - - -▲ extract; ■**- - - - -**■ vincristine sulphate) for 72 h incubation. Every point represents the mean of triplicate samples. Error bars represent the standard deviation.

Cytotoxicity effects of the extract towards HL-60 and NIH/3T3 cells were determined by measuring the cell viability using MTT assay after 72-hour treatment with the different concentrations of extract. The CD_50_ value was obtained from the plot between the concentrations of extract versus percent of cell viability. The value was used to describe the degree of cytotoxicity of the extract towards cell lines.  Figure 1 shows that CD_50_ of the extract for the HL-60 cells was 0.9 *μ*g/mL. The graph also gave the concentration of the extract that reduced 25% of the cell population (CD_25_) at 0.45 *μ*g/mL and for 75% reduction (CD_70_) the concentration was at 1.35 *μ*g/mL. However, the extract was less cytotoxic towards normal mouse fibroblast cell line NIH/3T3. The viability was not affected when treated with the extract at 15.0 *μ*g/mL concentration but the viability of the cells was reduced to 80% when treated at 30.0 *μ*g/mL (*P* < 0.05). In this study, vincristine sulfate, a commercial drug for the treatment of leukemia and multiple myeloma was used as a positive cytotoxic control compound [24]. Vincristine is a vinca alkaloid from *Catharanthus roseus* (Madagascar periwinkle) and the compound is known to be a mitotic inhibitor [25]. The CD_50_ of vincristine for HL-60 cell was 0.2 *μ*g/mL and 2.5 *μ*g/mL for NIH/3T3 cells (*P* < 0.05). The viability of NIH/3T3 cells was drastically reduced to about 50% for the concentration of the extract below 2.0 *μ*g/mL and remained at about 50% when the extract concentration was increased even at 30.0 *μ*g/mL (Figure 1). 

The extract was considered to possess a very strong cytotoxic activity towards HL-60 cells. The CD_50_ value of the extract was the same as the value of goniothalamin, a styrylpyrone derivative isolated from *Goniothalamus* species towards CEM-SS T-lymphoblastic leukemia cells which was reported by Ali et al. [22]. Extracts or compounds which demonstrated the CD_50_ value of 10–25 *μ*g/mL were considered to be weak in cytotoxicity while compounds with the CD_50_ value of less than 5.0 *μ*g/mL were considered very active. Those compounds or extracts that have intermediate value between 5.0 and 10.0 *μ*g/mL of CD_50_ value were classified as moderately active [6]. 

**Figure 2:**
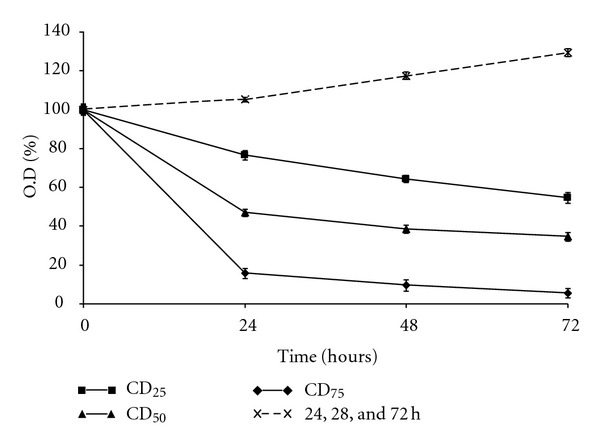
The growth curve of HL-60 cells after treatment with *C. cauliflora* extract at different cytotoxic doses (■**—**■ CD_25_, ▲**—**▲CD_50_; ♦**—**♦CD_75_: untreated X- - - -X) for 24, 48, and 72 h. Every point represents the mean of triplicate samples. Error bars represent the standard deviation.

The effects of extract at CD_25_, CD_50_, and Cd_75_ concentrations on the HL-60 cell proliferation was studied over a period of 72 hours. The absorbance at 570 nm wavelength after MTT assay was used as the indirect measurement number of viable cells. The cell proliferation rate was found to be significantly reduced with concentration dependence especially for the first 24 hours and further reduced at 48 and 72 hours (Figure 2). At CD_25_ concentration, the viable cells were decreased to 76.5% at 24 hours and further decreased to 64.3 and 54.5% at 48 and 72 hours, respectively (*P* < 0.05 with respect to the untreated cells at the same time). At CD_50_ concentration, the viable cells were reduced to 46.9% at 24 hours and decreased to 34.5% at 72 hours. The viable cells of the HL-60 cells treated at CD_75_ were decreased to 15.67% at 24 hours and further decreased to 9.5 and 5.4% at 48 and 72 hours, respectively (*P* < 0.05 with respect to the initial cell concentration). The proliferation rate of untreated HL-60 cell on the other hand was increased, as reflected in the OD values which were increased to 105.2, 117.5, and 129.3% at 24, 48, and 72 hours, respectively (*P* < 0.05 with respect to the untreated cells of initial cell concentration).

### 3.1. Mode of Cell Death in HL-60 Cells

**Figure 3:**
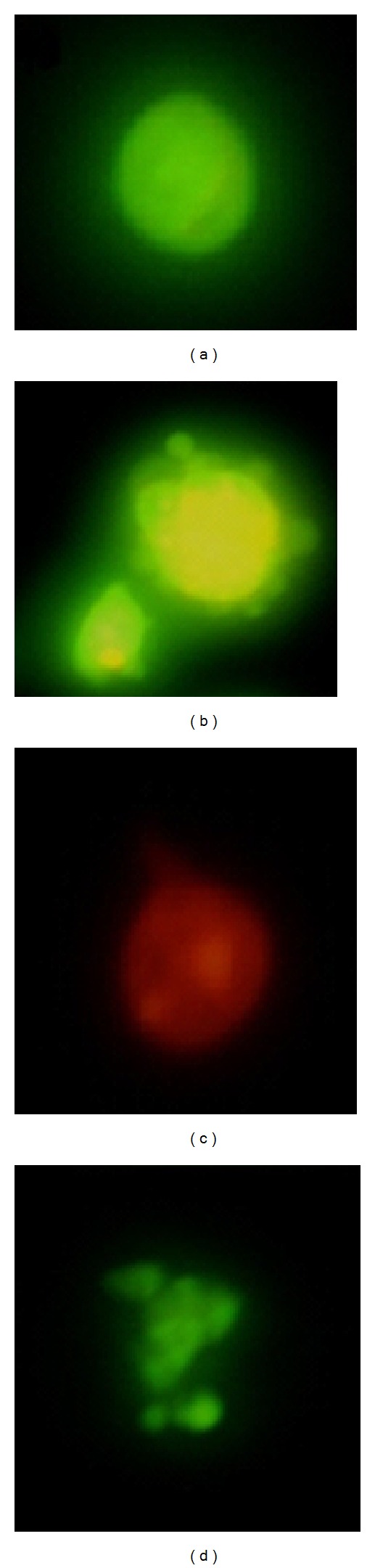
Morphological assessment of HL-60 cells treated with the extract of C*. cauliflora* for 24 h at CD_50_ after staining with acridine orange (AO) and propidium iodide (PI). (a) viable; (b) apoptosis; (c) necrosis; (d) apoptotic bodies (400x magnification).

 Acridine orange and propidium iodide dyes were used to differentiate viable, apoptotic, and necrotic cells under fluorescence microscope.  Figure 3 shows the intact viable cells (V), apoptotic (A), and necrotic cells after the HL-60 cells were treated with the extract at CD_50_ concentration for 24 hours. Selective permeability property of intact plasma membrane of viable cells allowed the acridine orange to enter the cell and was impermeable to the propidium iodide. The nucleus of viable cells were stained with green-orange when observed under fluorescence microscope whereas the plasma membrane of necrotic cells was no longer intact which allowed propodium iodide to enter and make the cells appear red [22]. The integrity of plasma membrane of apoptotic cells is still intact but the morphology of the cells had changed with blebbing plasma membrane and condensed nuclear chromatin [26–28].

**Figure 4:**
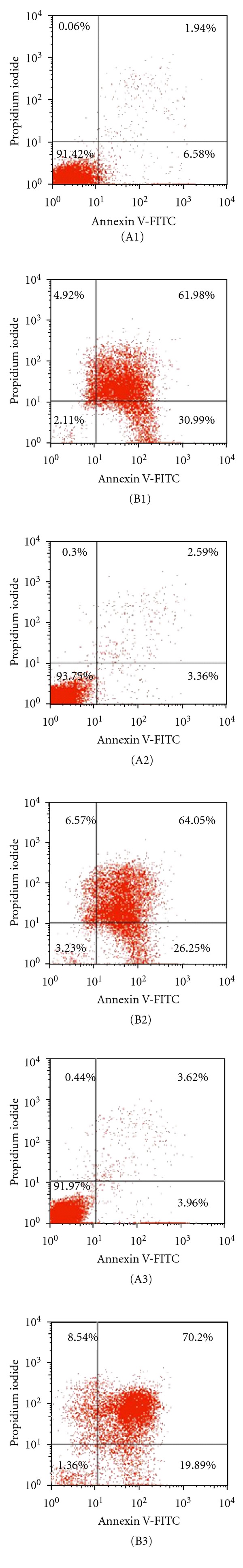
Flow cytometry analysis of HL-60 cells untreated (A1 = 24 h, A2 = 48 h, A3 = 72 h) and treated with the extract at CD_50_ (B1 = 24 h, B2 = 48 h, B3 = 72 h) stained with annexin V-FITC/propidium iodide (PI). Viable cells are in the lower left quadrant, early apoptotic cells are in the lower right quadrant, late apoptotic cells are in the upper right quadrant, and non-viable necrotic cells are in the upper left quadrant. Dot plots are a representative of 10,000 cells from a single replicate.

The proportion of HL-60 cells in the early and late apoptotic and necrotic cell death modes was determined after the cells were treated with extract at CD_50_ dose for 24, 48, and 72 hours. The treated cells were stained with annexin V/PI and approximately 10,000 cells were analyzed by using the flow cytometry. The analyzed cells were grouped into four quadrants (Figure 4). The quadrant at the bottom on the left represents the viable cells which do not take both dyes. While cells at the early stage of apoptosis were stained with annexin V due to high affinity to the externalized phosphatidylserine on the surface of plasma membrane which represented in the second quadrant at the bottom on the right. Cells that were in the late apoptosis were stained with both dyes which represented in the third quadrant at top right and cells in necrosis were stained only with PI which represented in fourth quadrant at the top left [23]. The proportion of HL-60 cells in the early apoptosis was 31.0, 26.3, and 9.9% after 24, 48 and 72 hours, respectively (*P* < 0.05 respective to untreated cells at the same time). For cells that were in the late apoptosis, the proportion was increased from 62.0% at 24 hours to 64.1 and 70.2% at 48 and 72 hours, respectively. Meanwhile, the proportion of the cells in the necrosis was 4.9, 6.6, and 8.5% at 24, 48, and 72 hours, respectively (*P* < 0.05 respective to untreated cells at the same time). For the untreated HL-60 cells, the percentages of cells that undergo necrosis were below 1% at 72 hours incubation time (Figure 4). 

## 4. Conclusion

This study had shown that methanolic extract of *C. cauliflora* whole fruit was very cytotoxic towards HL-60 cells and inhibits the cell proliferation. The extract induced the cells to die in majority through apoptotic cell death mode. However, the extract was found to be less cytotoxic towards 3T3/NIH cells. This observation is very important for future study on the isolation of cytotoxic compound(s) that are present in the extract. Furthermore, the *in vivo* antileukemic activity of the extract can be evaluated by using Balb/c leukemic mice induced with the WEHI-3B cells as reported by Alabsi et al. [29].
